# Who’s afraid of the political novel? An introduction

**DOI:** 10.12688/openreseurope.19915.1

**Published:** 2025-06-16

**Authors:** Ivana Perica, Aurore Peyroles

**Affiliations:** 1 Leibniz Centre for Literary and Cultural Research, Berlin, Berlin, Germany

**Keywords:** political novel, the politics of literature, novel, politics/the political

## Abstract

Our purpose in this introduction and in the research project The Cartography of the Political Novel (Caponeu), to which this collection belongs, is not to introduce a new category that complements – or opposes – other established genre designations such as the engaged novel or the thesis novel. Rather, it is about examining the relevance of the term ‘political novel’ anew. Why do we consider it important, both as a tentative genre and as a politically productive and dynamic social phenomenon, both in its past, its present and – predictably – in its future? While recognising the current scholarly interest in the inherently political nature of all writing, we argue that certain novels, unlike many others, benefit from being more directly designated as political novels.

Against Political Literature: In their provocatively titled book, *Contre la littérature politique* (see 1), six authors who are among the most far-left writers on the French literary scene express their distrust of a buzzword that has become ubiquitous in academic and media criticism – ‘political literature’. This rejection is not a defence of an apolitical literature that sits firmly in its ivory tower and is entirely devoted to art for art’s sake, but rather a criticism of the systematic use of an adjective that loses its meaning when it is associated with any form of literature that can be vaguely described as progressive and pretends to make the invisible visible and the unheard audible. It shows how much the term ‘political literature’ has come into fashion – and how problematic it is.

In the present context, the ‘political novel’ is no longer one that “does not dare to speak its name”, as Susan Rubin Suleiman points out is the case with the “ideological novel” ( 2 on page 4), but, on the contrary, it is one that proclaims itself and is praised as such. Far from being just the latest outcry of sales-oriented self-promotion, novels such as *Kissani Jugoslavia* (2014) by Pajtim Statovci, *Our Life in the Woods* (2017) by Marie Darrieussecq, *Clavicle* (2017) by Marta Sanz, *Assembly* (2021) by Natasha Brown and *Ada’s Room* (2021) by Sharon Dodua Otoo not only testify to the continuing relevance of the novel but also mark a politicisation of literature that is not unprecedented in the history of novel writing itself. As the above titles suggest, contemporary literature is revisiting various subgenres and literary styles and may also be revitalising certain political legacies to meet the needs of different groups of readers. Literary movements and aesthetic legacies as diverse as high modernism, critical realism, *littérature engagée*, autofiction and various forms of utopian and dystopian writing are emerging anew.

Our purpose in this introduction and in the research project The Cartography of the Political Novel (Caponeu), to which this collection belongs, is not to introduce a new category that complements – or opposes – other established genre designations such as the engaged novel or the thesis novel. Rather, it is about examining the relevance of the term ‘political novel’ anew. Why do we consider it important, both as a tentative genre and as a politically productive and dynamic social phenomenon, both in its past, its present and – predictably – in its future? While recognising the current scholarly interest in the inherently political nature of all writing, we argue that certain novels, unlike many others, benefit from being more directly designated as political novels. This leads us to reformulate a famous question about works of art: “There are political novels – how are they possible?” ^1^

*The political novel*. What does this term imply? What reading does it require? Which critical tools does it rely on? And above all, what difficulties does it pose? We want to take a critical look at a designation that raises numerous methodological and theoretical problems and that appears all the more problematic – but also, we hope, fruitful – because the definition of the political is continually changing, constantly including new objects at the risk of excluding others. Rather than being a universal category, the designation ‘the political novel’ is in fact highly polemical. In trying to determine the relevance of an expression that is both consensual and problematic, we are confronted with Stuart Hall’s statement about popular culture. To rephrase it for our purposes, we could say that we “have almost as many problems with ‘the political’ as we have with the ‘novel’. When you put the two terms together, the difficulties can be pretty horrendous” ( 4 on page 72; qtd. in 5 on page 1). ^2^ So how should we proceed when each component of our research object proves problematic: the – political – novel?

Firstly, the novel. Its immense formal plasticity, bordering on indeterminacy, is one of the characteristic features of the novel as a genre. It is the “genre of what is without genre” ( 6 on page 29; see also 7), “the other of all genres” ( 8 on page 78) or even the “end” ( 9 on page 137) of the genre. In the words of the French writer Pascal Quignard, the novel “degenerates” ( 8 on page 78). Even the somewhat intuitive consensus on the novel as a long fictional narrative in prose is (once again) wavering. In France, for example, the Prix Goncourt for the best novel of the year recently has been awarded to books that completely dispense with the fictional mode and the voluntary suspension of disbelief. ^3^ The fact that such books are still labelled as novels is less an indication of their genre characteristics than a signal of their role in the market and, accordingly, the size of the readership they appeal to. As Franco Moretti emphasises, the novel is “always commodity and artwork at once” ( 10 on page ix; see also 11 on page 125).

If the noun ‘novel’ is so imprecise, does the adjective ‘political’ capture the salience of the literary phenomenon whose characteristics we are attempting to outline here? Since its modern conception, the novel has been celebrated for its ability to integrate different forms of expression and praised for its “participation in historical becoming and in social struggle” ( 12 on page 331) – or criticised for potentially “foment[ing] ambitions for social advancement that would threaten the established order” ( 13 on page 144). Through its ability to resonate a polyphony of voices, the novel has not only expressed the aspirations of the “intellectual proletariat” ( 13 on page 144) but has also demonstrated its adaptability across ideological boundaries (left and right, grassroots and authoritarian, subversive and constructive) and within different political systems, historical epochs and social classes. This formal and ideological flexibility has cast the novel an emblem of modernity – a genre that captures the irreversible fragmentation and the accompanying sense of “transcendental homelessness” ( 14 on page 41) experienced by its subjects. The novel is thus the literary form that takes up a demythologised world given over to politics and its divisions.

In this way, it maintains a privileged connection to politics. It gives voice (or can give voice) to a multiplicity of viewpoints that question dogmatic and authoritarian positions. This is the core of Jacques Rancière’s “politics of literature” ( 15), which remains one of the guiding concepts of literary studies. By decoupling the politics of literature from “the politics of writers and their commitments” or “the way they represent social structures or political struggles” ( 16 on page 53), Rancière argues that literature makes politics *as literature*. Only as literature, and not as a proxy of other sorts of politics, is literature able to do politics, that is, it “makes visible what had no business being seen, and makes heard a discourse where once there was only place for noise” ( 16 on page 30). It thus expands the range of themes, entities and events worthy of representation and discussion. Because of its ability to make a polyphony of voices resonate far beyond the authors’ original or explicit intentions, and because of the attention to the world that this genre displays, the novel is always-already involved in sustaining or reshaping the “distribution of the sensible” ( 17). As such, it is considered by many theorists to be an essentially democratic genre (see 18 and 19). This would lead to the conclusion that the novel is inherently political. And this is precisely the point where the problem with the ‘political novel’ arises. The adjective ‘political’ not only becomes superfluous, making ‘the political novel’ a tautology that merely emphasises what is already inherent. It also conflates the specificity of literature with the exigency of politics.

We believe that this disengagement of literature from traditional notions of authorial intention and the politics of representation, while intellectually compelling, poses two major challenges. First, it leaves open the question of how to distinguish a political novel from an apolitical novel (is a decidedly apolitical novel even possible?). Secondly, it ignores the fact that some novels are indeed coded and decoded as political precisely because they correlate with the politics of their authors and/or are orientated towards or negotiate broader social and political struggles. Rancière’s separation of the politics and activism of writers from the politics of their literature undoubtedly represents a pivotal point in the development of the classical concept of engaged literature. While this shift expands the understanding of literary potential, it also obscures the meaning of politics itself.

This difficulty in grasping and circumscribing politics is reinforced by the development of the concept of ‘the political’ in both political and literary theory. Detached from the traditional understanding of politics as organised practices represented by parties, trade unions, professional politicians or entire regimes (including democracy), ‘the political’, since its emergence in the 1980s, and especially in literary theory since the 2000s, has stood for a critical questioning of these practices (see 20; 21, esp. pages 227–228; 22; 23). It aims to break through their taken-for-grantedness and reshape politics from the perspective of the social margins, the oppressed subjects, the geographical peripheries and other invisible dimensions of the social. For example, Irving Howe’s *Politics and the Novel* (1957) was praised in the late 1950s for defining the political novel as a novel “in which political ideas play a dominant role or in which the political milieu is the dominant setting” ( 24 on page 471; see 25). In the 1990s, however, this definition was scrutinised for its limitations and provoked questions such as that posed by William Sharpe: “But whose politics, whose novel, whose nerves?” ( 26 on page 207). Howe’s focus on ideas, contexts and settings neglected what the discourse of the 1990s had already labelled “the politics of everyday life” ( 26 on page 207). Sharpe concludes his critique with a quote from Adrienne Rich: “The moment a feeling enters the body / is political. This touch is political” ( 26 on page 207). What is considered political evolves and resonates differently in different geographical and historical contexts. The clear-cut framework of politics – centred on the state, nation or class – is increasingly giving way to a broader, more fluid and ambiguous understanding of ‘the political’.

Recent scholarly endeavours increasingly show how a broader conceptualisation of ‘the political’ enriches our understanding of literature, both historical and contemporary. Drawing on thinkers such as Jacques Rancière, Claude Lefort and Pierre Rosanvallon, these studies historicise the notions of politics/the political in the context of the twentieth century and the present. Focusing primarily on German-language literature, particularly contemporary literature ( *Gegenwartsliteratur*), Immanuel Nover and Stefan Neuhaus provide a valuable framework for analysing the evolving meaning of ‘politics’ and ‘the political’ across linguistic, regional and historical boundaries. In *Das Politische in der Literatur der Gegenwart* (The Political in Contemporary Literature), they argue that contemporary literature no longer responds to political paradigms in the same way as Cold War-era literature, which often took clear positions in favour of or against identifiable ideologies. Instead, contemporary works engage in political discourse through strategies that are interpreted within the literary field as contributions to political dialogue *sui generis* ( 27 on page 12). In defending the political character of contemporary German literature, Neuhaus and Nover criticise what they call a “structural error of thought” ( *struktureller Denkfehler*, 27 on page 5) in literary studies, namely the persistent belief that post-war literature ( *Nachkriegsliteratur*) can indeed be classified as political, while contemporary literature is considered insufficiently politically engaged. In doing so, they join a broader international theoretical consensus which recognises the presence – and often the subtle traces – of ‘the political’ in areas not traditionally classified as political, thus challenging conventional boundaries and extending the reach of the politics of literature. ^4^

However, the growing importance of the concept of ‘the political’ and its increasingly broad application do not necessarily lead to a clear or even practical definition of political literature (or the politics of literature, for that matter). Michael Navratil notes that “no strict definition can adequately define what political literature actually is” ( 29 on page 196), and Neuhaus and Nover similarly concede that “the opening from politics to the political makes it impossible to provide a clear definition that can separate political from non-political texts” ( 27 on page 6). If the ubiquitous use of the substantivised adjective the ‘political’ in academic research, literary criticism, publishing and the media emphasises the high status of literature that touches on the political, this prominence does not imply a contemporary revival of the interplay between ‘politics and the novel’, as described by Irving Howe and characteristic of twentieth-century literary landscapes. Contemporary literature often positions itself as a representative voice for those neglected by political systems, appealing to empathy rather than questioning the structural causes of invisibilisation. The political dimension of such novels is seen in their ability to “repair the world” ( 30), fostering connections and creating a sense of a shared universe. This approach tends to reduce ‘the political’ to a consensus-oriented ethos that avoids conflict – a premise that invites criticism. This is precisely what the authors of the provocatively titled volume *Contre la littérature politique* argue against. The target of Alferi and colleagues’ critique is not political literature *per se*, but the very dilution of the concept of ‘the political’ (see 1). They argue in favour of a literature that deals more directly and radically with the conflicts that permeate the social world. Building on this critique and looking not only at the French but at various local contexts in which the political novel currently circulates – as a brand, as a negative designation by a political regime or as a feature of the seemingly long-gone world of dissident writing – the question arises as to who determines which novels are considered political and, more importantly, on the basis of which definition of politics.

This questioning also allows us to scrutinise the assumed association of politics with progressiveness. Political novels do not inherently or exclusively promote progressive agendas. They can also reflect regressive and reactionary forces – whether covertly or overtly anti-egalitarian – and thereby legitimise forms of oppression, violence and exclusion. In essence, the acclaimed redistribution of the sensible does not necessarily promote more equality and freedom; it can also reinforce contrary tendencies. None of the texts in this collection are devoted to a novel epitomising an ideology that could be labelled as reactionary, nor to a novel supporting an existing political regime and its established order or any emerging neo-imperial constellations. The novels analysed here are instead highly critical. They denounce the immediate context in which they were written or raise fundamental questions about the political organisation of modern and contemporary societies, republics and democracy. This ‘omission’ is certainly due to the – fortunate, we add – peculiarity of the literary field, which for decades has been predominantly oriented towards inclusive and progressive writing practices and has displayed an emphatically critical view of traditions and regimes based on the exclusion and erasure of dissensual genealogies. In this sense, we acknowledge that there is an urgent need to turn our attention to contemporary literary production of the right and far-right spectrum, which seems to collaborate productively with political movements that take place outside the purely literary sphere (see 31). The political is not limited to what we would like it to be, a force that strengthens the collective and promotes emancipation.

Another critical problem with the inflationary use of the substantivised adjective ‘the political’ is the risk of its dehistoricisation – the indiscriminate application of ‘the political’ to different historical contexts without adequately taking into account the particular political, social, cultural, institutional and other circumstances that mark the appearances of political novels across times and spaces. In other words, it is important to examine how the politics of a certain geographic area or a historical era, including its ideological underpinnings, influence the emergence of ‘the political’ (as that other part of ‘politics’ that challenges its forms of rule) and how novels address, reflect and embed these dynamics in their plots and narrative structures. In the twentieth and twenty-first centuries, the relationship between politics and literature in Europe has often been dynamic and at times extreme. In some cases, political regimes have suppressed dissident, subversive and even ‘degenerate’ novels, ^5^ while in other cases, novels that have challenged or rejected political authority have found unexpected public resonance and gained considerable political prominence. Within this complex relationship, novels have contested politics so thoroughly that even the most private, hidden or seemingly apolitical aspects of life have become fertile ground for the novel to assume the mantle of the political novel. ^6^ At the same time, however, there have also been cases where literature and politics have entered into a mutually supporting dialogue, assisting and influencing each other in profound ways.

The diversity of (political and economic) contexts, the different interpretations of ‘the political’ and the ongoing debate about what should be labelled a novel all contribute to the inherent instability of the political novel. This is all the more true as the political novels presented in the Caponeu Digital Map lack any recurring formal features. If the political novel were a straightforward genre category, its strict definition would require the identification of common formal or thematic elements as well as specific references to a particular time or cultural and geographical context. Instead, the novels analysed in our project resist conforming to existing norms or paradigmatic texts that can confirm or deny their classification into a single category. In this respect, the political novel stands in stark contrast to what Suleiman calls “authoritarian fictions” ( 2), i. e. fictions characterised by repetitive structures and narrative devices (exemplarity, coming-of-age and antagonistic structures, redundancy) that become tedious across different works. It would be difficult to find such repetitions, whether formal or thematic, in the novels discussed in this collection. “It is one thing to say that a novel produces political effects, and another to assume that it shares structural features with other novels that might best be treated collectively under the label of the political novel”, Zvonimir Glavaš observes ^32^. The versatility of the political novel certainly sheds a different light on Robert Frost’s couplet quoted by Jonathan Culler in his introduction to literary theory: “We dance round in a ring and suppose, / But the Secret sits in the middle and knows” ( 33 on page 55). The characteristics of the political novel, however, are not so predetermined that they ‘sit in the middle’, just waiting to be discovered by diligent philological experts. To refer to Paul Kincaid’s analysis of the challenging genre of science fiction to describe the political novel:

What constitutes the warp and weft of [the political novel], therefore, is endlessly subtle and intricate, made up at times of more things than we can readily identify. Which is why what makes [the political novel] is so hard to pin down, but what is [the political novel] is so easy to recognize. ( 34 on page 417)

Much like the novel as a genre, which is best understood as “variable rather than constant” ( 35 on page 272), the political novel can be seen as a series of works that merely share certain family resemblances.

The impossibility of constituting the political novel as a literary genre should not deprive us from the possibilities and fruitful analyses that this category opens up. We are convinced that it is useful, not only in academia, but ideally beyond literary specialists. Special attention to novels that not only touch on the political but also deal more directly with certain political themes and issues seems necessary to us – on the one hand, to avoid a dilution of political literature as in Rancière’s politics of literature and, on the other hand, to still find *a political approach* to novels that are not explicitly committed novels or in which political figures and events do not “dominate the plot” ( 11 on page 3). We consider the term ‘political novel’ as a comprehensive category which encompasses both literature that is explicitly intended to make a political statement or serve as an ideological tool – an aim that is often criticised as a reprehensible form of instrumentalisation, even though some contemporary authors express a desire to influence social dynamics through their writing (see 28 on page 858) – as well as novels that achieve their political incisiveness through subcutaneous, micropolitical, even “opaque” ( 36) work with omissions, tacit resonances and silences on topics traditionally perceived as political. The political novel is therefore best understood as an umbrella term that encompasses various literary phenomena in which politics and the novel overlap.

As an interpretative framework, the ‘political novel’ echoes the endeavour to uncover the “political unconscious” ( 9) of a literary text but also goes beyond this to focus on the ways in which a political novel reflects on and engages with political concerns, as well as the ways in which its particular politics are perceived by different readers and audiences. It presupposes that the political dimension of the novel does not lie exclusively in the declared intentions of the author but can arise independently of these intentions and possibly in opposition to them, and sometimes only becomes clear in retrospect. Of central importance here is the contextual positioning of the novel within the circumstances of its creation and reception, the use of certain literary forms to calibrate political themes and the readings that are promoted or discouraged by these forms.

Using the category of the political novel, we aim to promote and develop a historically nuanced, geographically and ideologically fine-tuned approach that encompasses the full span of the relationship between the novel and the political: the broader political frameworks that shape and intervene in the normative world in which a novel is produced, and the political novel as a mode of negotiating politics, a way of (de)instituting it from a critical, parallax, even revolutionary perspective. If the former is the “path of the object” ( 9 on page ix), namely the historical constitution of “the things themselves”, as Fredric Jameson famously puts it, then the political novel emerges as “the path of the subject” ( 9 on page ix). It consists of a specific perspectivisation of an object, an invitation, even an instruction, to see in a certain way. This invitation does not necessarily come from the authors, but also from editors, critics as well as professional and empirical readers, ^7^ sometimes belatedly and in contexts that differ significantly from those in which the novel was originally written.

This integrative approach that takes both paths into account allows for a more comprehensive understanding of the fruitful interplay between social and literary forms. It examines the political, economic and social paradigms that define the aesthetic coordinates within which a novel is created and explores how these paradigms shape its literary forms – in other words, how “the sociality of form” ( 37 on page 49) manifests itself in the work. In sum, we argue that the term ‘the political’, as it is currently used, needs to be revived not only as the opposite of ‘politics’ but as a marker of *politicisation*. Only by considering the specificity of certain political novels in terms of their potential to politicise and thus accelerate the political realm ( 38 on page 76) or to problematise the consensus that promotes the status quo can ‘the political novel’ retain its analytical utility. By giving the adjective political the strongest possible meaning, we also want to examine how political novels contribute to our understanding of politics. In what ways might they redefine it? How do they intersect with other discourses that are collectively experienced or remembered as political transformations? In particular, how do they reflect broader shifts in perception that are simultaneously reshaping our political forms of coexistence? If political novels capture or articulate critical turning points, shift perspectives and resonate with new and emerging political and aesthetic regimes, through what formal, stylistic or discursive strategies do they achieve this?

By focusing on this dynamic process, we aim not so much to find a definitive definition as to forge an interpretative and praxeological approach that emphasises social (and economic and cultural) dimensions – such as pragmatic interests, ideological aspirations, strategic provocations and marketing strategies (see 40) – over purely narratological or thematic analyses. We therefore propose the following:

The political novel is a set of practices through which a novel is coded and decoded as political within a specific constellation of circumstances – epistemological, historical, literary, national, political and linguistic. It is identified less as a particular genre than as a dynamic process of becoming and transformation that involves the reappropriation, repositioning and reorganisation of different circumstances. ^8^

At this point in our reflections, the political novel emerges less as a genre defined by specific norms and more as an interpretive framework – a lens through which one engages with a text as a political object and makes it the subject of a debate (beyond purely literary analysis) that can raise awareness or simply provoke discussion. ^9^ Neither this introduction, nor this collection, nor the broader work of The Cartography of the Political Novel in Europe aim to establish a definitive classification of the political novel as a genre. Instead, the collection seeks to initiate a dialogue about an alternative and, in the most constructive sense, “disposable” ( 41 on page 110) canon of works that have been, are or could be read and discussed as political novels. Without aspiring to create a comprehensive and definitive archive of past or present political novels, this collection invites readers to approach novels as political experiences. Such experiences evoke and appeal to particular emotions, foster (or undermine) specific loyalties or suspicions and, in some cases, explicitly advocate for various forms of emancipation. Ultimately, this is an invitation to read novels politically.

The collection is divided into two parts. The first consists of texts that reflect on the category of the political novel in general, containing questions raised from different angles that emphasise its current importance: What forms does (can) the political novel take (Zrinka Božić ^42^)? What relationships does it establish (or not) with its readers (Zvonimir Glavaš ^32^)? How does it shape our perceptions, reinforcing or criticising the hegemonic manipulations that forge them? Is it able to open up new emancipatory horizons? (Tomasz Mizerkiewicz ^43^)? To what extent do feminist readings contribute to shaping or renewing this category (Mirela Dakić ^44^)? Beyond the examples and theories mentioned, these contributions have the merit of considering the political novel as a framework for analysis that sheds new light on classical questions of narratology and literary theory. This is evidence that ‘the political novel’ invites us to think afresh about questions that recur whenever we take an interest in the novel genre.

The second part is devoted to case studies. Proposed readings of Vladan Desnica’s novel *Zimsko ljetovanje* ( *The Winter Summer Vacation*, 1950), Samuel Beckett’s *Molloy* (1951), Michał Witkowski’s *Lubiewo* (2005) and Pierre Michon’s *Les Onze* ( *The Eleven*, 2009) take up the challenge set at the end of this introduction, namely to read novels politically that are not systematically or primarily considered as such. The contributions authored by Marina Protrka Štimec ^45^, 46 Paul Stewart, Błażej Warkocki and Nenad Ivić ^47^ testify to the analytical relevance of the category of the political novel, as they often focus on aspects that have previously been neglected in the reading of the works mentioned. They show what an actualising and politicising reading can contribute to the interpretation of a novel.

The spectrum of texts identified as political novels by the authors of this collection and of the Caponeu members in general is astonishingly diverse. It includes realist novels, fragmented narratives with autobiographical elements, self-reflexive and meta-reflexive fictions, first- and third-person perspectives and, finally, both widely accessible works and niche publications. Some of these texts are explicitly orientated towards Jean-Paul Sartre’s philosophy of engagement, while others actively reject it; some are assertive, others are characterised by ambiguity and contradiction, revealing a broad spectrum of differences. However, this diversity by no means guarantees that all the political novel’s intentions and the forms that it can take are represented. While there are some significant works included, the texts traditionally emphasised by theorists of the political novel – those that have formed the canonical core of the genre over the last century – are notably absent.

The heterogeneity of texts and approaches in this collection does not represent all the possibilities contained in the concept of the political novel, and many modalities remain unconsidered. Therefore, rather than smoothing over the many differences between these texts and the respective analytical frameworks, the individual contributions examine how they can be interpreted through a political lens – whether they are traditionally perceived as politically engaged or typically analysed within a political or ideological framework. This reflects our collective research aim as scholars – to interrogate what the term ‘the political novel’ means today, in 2025, and how this understanding relates to themes that have historically been excluded or marginalised from mainstream political discourse but which have now shifted to the centre of contemporary analyses.

## Ethics and consent

Ethical approval and consent were not required.

## Data Availability

No data associated with this article.

## Footnotes

^1^ In the original: “There are works of art – how are they possible?” ( 3 on page 9).

^2^ In the original: “I have almost as many problems with ‘popular’ as I have with ‘culture’. When you put the two terms together, the difficulties can be pretty horrendous.”

^3^ The winners of the Prix Goncourt in 2017 and 2022 are good examples of this shift. *L’Ordre du jour* ( *The Order of the Day*, 2017) by Éric Vuillard is described as a *récit* and recounts several historical episodes from the beginnings of the Third Reich in the 1930s. *Vivre vite* ( *Live Fast*, 2022) is an autobiographical story in which Brigitte Giraud describes the circumstances surrounding the accidental death of her husband.

^4^ Less than five years after Neuhaus and Nover’s publication, the consensus on the political nature of contemporary German-language literature has become so strong that hardly anyone would argue that this line of cultural production is apolitical, or feel the need to justify its politics in comparison to post-war writing – and yet its political nature is now recognised in the various calibrations of ‘the political’, not ‘politics’. See 28.

^5^ To illustrate: Norman Manea’s *Plicul negru* ( *The Black Envelope*, 1986) was subject to the strict censorship of Romania’s communist regime, which eventually forced the author to leave the country for good in 1986; Pier Paolo Pasolini’s literary (and cinematic) work is famous for its not only subversive but also visionary potential in the years before the gay and lesbian movement, and his unfinished novel *Petrolio* ( *Petrol*, published posthumously in 1992) is sometimes associated with the still unexplained motives for his assassination in 1975.

^6^ Milan Kundera’s *Žert* ( *The Joke*, 1967) serves as a prime example. The story of how a student’s private joke turns his life upside down reflects the political realities of post-1968 Czechoslovakia. Written in Prague in 1965, the book’s publication was delayed by censorship until 1967, only to be banned in 1968 after the Soviet Union crushed the Prague Spring. The author eventually left Prague and Czechoslovakia in 1975 and settled in Paris.

^7^ We prefer not to contrast professional readers with non-professional audiences, but rather with empirical readers, i. e. the readers we really are in our everyday reading habits and practice (see 39 on page 165).

^8^ An earlier, similar version of this explanation was included in the application proposal of the Caponeu project (Horizon Europe, Grant agreement ID: 101094658).

^9^ Our research project involves several book clubs and reading events (notably in Berlin, Cambridge, Poznań and Zagreb), providing an opportunity to see to what extent the proposed novels, presented as political, provoke debate and discussion.

## References

[1] Alferi P Kaplan L Quintane N : Contre la littérature politique.Paris: La Fabrique,2023. Reference Source

[2] Suleiman SR : Authoritarian fictions. The ideological novels as a literary genre. New York: Columbia University Press,1983. Reference Source

[3] Lukács G : Heidelberger Ästhetik (1916–1918).In: *Georg Lukács Werke. Vol. 17. Frühe Schriften zur Ästhetik II.*Neuwied: Luchterhand, 1974. Reference Source

[4] Hall S : Notes on deconstructing the popular.In: Imre Szema and Timothy Kaposy (eds.). *Cultural Theory - An Anthology.*Oxford: Blackwell, 2011;72–81. Reference Source

[5] Philips D Shaw K, (eds.) : Literary politics. The politics of literature and the literature of politics.New York: Palgrave Macmillan, 2013. 10.1057/9781137270146

[6] Rancière J : Mute speech: literature, critical theory, and politics. Translated by James Swenson. New York: Columbia University Press,2011. Reference Source

[7] Cunningham D : Genre without genre: Romanticism, the novel and the new. In: *Radical Philosophy.* 2016;196:14–27. Reference Source

[8] Quignard P : La déprogrammation de la littérature.In: *Le Débat*.1989;54:77–88. Reference Source

[9] Jameson F : The political unconscious. Narrative as a socially symbolic act. Ithaca, NY: Cornell University Press,1981. Reference Source

[10] Moretti F, (ed.) : On the Novel.In: *The Novel. Vol. 1. History, Geography, and Culture.*Princeton and Oxford: Princeton University Press, 2006;ix–x. Reference Source

[11] Santin BM (ed.) : The Cambridge companion to the twentieth-century American novel and politics. Cambridge: Cambridge University Press,2023. 10.1017/9781009030274

[12] Bakhtin MM : The dialogic imagination. Edited by Michael Holquist. Translated by Caryl Emerson and Michael Holquist. Austin: University of Texas Press,1981. Reference Source

[13] Sapiro G : The sociology of literature. Translated by Madeline Bedecarré and Ben Libman. Stanford: Stanford University Press,2023. 10.1515/9781503637603

[14] Lukács G : The theory of the novel. A historico-philosophical essay on the forms of great epic literature.Translated by Anna Bostock. Cambridge: The MIT Press, 1971 (1920). Reference Source

[15] Rancière J : The politics of literature. Translated by Julie Rose. Cambridge and Malden: Polity,2011. Reference Source

[16] Rancière J : Disagreement: politics and philosophy. Translated by Julie Rose. Minneapolis: University of Minnesota Press,1999. Reference Source

[17] Rancière J : The politics of aesthetics: the distribution of the sensible. Translated by Gabriel Rockhill. London: Bloomsbury Academic,2004. Reference Source

[18] Wolf N : Le Roman de la démocratie. Paris: Presses universitaires de Vincennes,2003. Reference Source

[19] Servoise S : Parole d’écrivain et crise de la représentativité. In: *Revue critique de fixxion française contemporaine.* 2013; (6). 10.4000/fixxion.7926

[20] Bedorf T Röttgers K, (eds) : Das Politische und die Politik. Berlin: Suhrkamp,2010. Reference Source

[21] Lefort C : The permanence of the theologico-political?In: *Democracy and Political Theory.*Translated by David Macey. Cambridge: Polity, 1988;213–255. Reference Source

[22] Marchart O : Politics and the political: genealogy of a conceptual difference.In: *Post-Foundational Political Thought: Political Difference in Nancy, Lefort, Badiou and Laclau.*Edinburgh: Edinburgh University Press, 2007;35–60. Reference Source

[23] Mouffe C : Politics and the Political.In: *On the Political.*London and New York: Routledge, 2005;8–34. Reference Source

[24] Schoeck R : Review of Irving Howe’s *Politics and the novel* . In: *Review of Politics.* 1959;21(2):470–472.

[25] Howe I : Politics and the novel. New York: Horizon Press,1957. Reference Source

[26] Sharpe W : Review of Irving Howe’s *Politics and the novel* . In: *Polit Sci Q.* 1993;108(1): 206–207. Reference Source

[27] Neuhaus S Nover I, (eds.) : Das Politische in der Literatur der Gegenwart.Berlin and Boston: De Gruyter, 2019. Reference Source

[28] Spoerhase C Vogel J : Gegenwartsliteratur als Herausforderung des Literarischen. In: *Deutsche Vierteljahrsschrift für Literaturwissenschaft und Geistesgeschichte.* Stuttgart: Springer,2023;97:857–864. 10.1007/s41245-023-00240-7

[29] Navratil M : Politisches Schreiben: Kein Klärungsversuch.In: *Kontrafaktik der Gegenwart. Politisches Schreiben als Realitätsvariation bei Christian Kracht, Kathrin Röggla, Juli Zeh und Leif Randt.*Berlin and Boston: De Gruyter, 2022;195–204. Reference Source

[30] Geffen A : Repair the world: French literature in the twenty-first century. Translated by Tegan Raleigh. Berlin and Boston: De Gruyter,2024. Reference Source

[31] Neurechte Literaturpolitik. Research project led by Torsten Hoffmann, University of Stuttgart (1 Sept. 2023-31 Aug. 2026). Reference Source

[32] Glavaš Z : “From Charlemagne to the Title of the King”: Political novel between estrangement and recognition [version 1; peer review: awaiting peer review].2025;5:155. Reference Source 10.12688/openreseurope.19920.1 PMC12699207 41394320

[33] Culler J : Literary theory: a very short introduction. Oxford: Oxford University Press,1997. Reference Source

[34] Kincaid P : On the origins of genre. *Extrapolation* .2003;44(4):409–419. 10.3828/extr.2003.44.4.04

[35] Tynianov Y : On literary evolution. In: *Permanent evolution. Selected essays on literature, theory and film.* Translated by Ainsley Morse and Philip Redko. Boston: Academic Studies Press,2019 (1927);267–282. 10.2307/j.ctv1zjg7wx.17

[36] Glissant É : For opacity. In: *Poetics of Relation* . Translated by Betsy Wing. Ann Arbor: The University of Michigan Press,1997;189–194. Reference Source

[37] Kornbluh A : The order of forms: realism, formalism and social space. Chicago and London: The University of Chicago Press,2019. Reference Source

[38] Brown W : Edgework: critical essays on knowledge and politics. Princeton and Oxford: Princeton University Press,2005. Reference Source

[39] Felski R : The limits of critique. Chicago and London: The University of Chicago Press,2015. Reference Source

[40] Bourdieu P : Outline of a theory of practice. Translated by Richard Nice. New York: Cambridge University Press,1977. Reference Source

[41] Jameson F : The aesthetics of singularity. In: *New Left Review* .2015;92:101–132. Reference Source

[42] Božić Z : Rethinking the politics of form: The strange case of the political novel [version 1; peer review: awaiting peer review].2025;5:125. Reference Source 10.12688/openreseurope.19916.1 PMC12181763 40547538

[43] Mizerkiewicz T : Perceptions, cartographies, and ‘cartographies of time’ in the political novel [version 1; peer review: 1 approved].2025;5:137. Reference Source 10.12688/openreseurope.19924.1 PMC12308170 40741234

[44] Dakić M : What is a novel in the political novel? The perspectives of contemporary feminist theory [version 1; peer review: awaiting peer review].2025;5:156. Reference Source

[45] Protrka Štimec M : “Perché i xe bestie?!” Politics, race and exclusion in Vladan Desnica’s The *Winter Summer Vacation* [version 1; peer review: 1 approved with reservations].2025;5:135. Reference Source

[46] Stewart P : Fundamental operations of the political in Beckett’s *Molloy* [version 1; peer review: awaiting peer review].2025;5:141. Reference Source 10.12688/openreseurope.19926.1 PMC12280865 40697423

[47] Ivić N : Is Pierre Michon’s *The Eleven* a political novel? [version 1; peer review: awaiting peer review].2025;5:144. Reference Source 10.12688/openreseurope.19922.1 PMC12398675 40895217

