# Supplementary material for: Who’s afraid of the political novel? An introduction
Source: Open Res Eur. 2025 Jun 16;5:161. [Version 1] doi: 10.12688/openreseurope.19915.1 (PMC12409254; doi:10.12688/openreseurope.19915.1)
Supplement: Peer review history

# Reviewer report: Reviewer response for version 1

**DOI:** 10.21956/openreseurope.21543.r56094

**Published:** 2025-09-03

**Authors:** Lucie Amir

**Affiliations:** 1 Université de Grenoble Alpes, France, France

The essay *“Who’s Afraid of the Political Novel ? An introduction",* serves as an introduction to a collection dedicated to the relationship between literature and politics. It forms part of a European project focused on mapping the “political novel.”

 Acknowledging the contemporary inflation of the label “political” in literary criticism, it proposes to wager on the conceptual fruitfulness of the category and to delineate the features of a "political novel". The authors suggest conceiving it not as a genre, but rather as an approach to reading novels — a "politics of literature" to borrow a conceptualization widely used in Francophone academic contexts (Belgium, Canada, France), though barely mentioned in the essay. They thereby introduce a series of case studies that explore the different aesthetic realities encompassed by the label “political novel”.

 The article stands out for its undeniable thematic and theoretical relevance. Its starting point is accurate: the term “political” has become ubiquitous in critical discourse, often underpinning vague and overused formulas (“political novel,” “politics of literature”); at the same time, a significant number of recent publications already signal the emergence of a new critical moment — one that is both more militant and more reflexive regarding the political power of literature. The essay *Contre la littérature politique* is symptomatic of this trend, but it is actually only one among many publications released in 2023–2024, particularly in France, that the article (which seems rather centered on the Francophone world) could have addressed (Huppe 2023; Alferi et al., 2024; Andras & Harchi 2024; Lucbert 2024; Coste 2024).

 The article’s main weakness lies precisely in how it justifies the chosen category. The proposal to treat the *“*political novel” as an interpretive framework to be tested and evaluated is indeed stimulating, and the authors’ intention to ground their approach in an empirical category is understandable. However, the very phrase “political novel” resists their project at the grammatical level: they wish to move away from rigid labels and approach the "political novel" as a way of engaging with texts, yet the concept they adopt reintroduces a classificatory logic. In other words, while the authors repeatedly state that they do not aim to define a genre, it is difficult to see how the concept of the *political novel* can escape that logic — especially since the article implies that some novels are more deserving of the label “political” than others. As a result, the article maintains a certain ambiguity throughout in its scholarly positioning: is it proposing to study how novels are politicized, as it claims repeatedly, or is it endorsing a particular theory of the political novel and promoting a reading of texts through that lens? The choice of the term “political novel” which seems intended to support both aims at once, ends up obscuring the project.

 In any case, the article would benefit from positioning itself more explicitly in relation to the concept of “politics of literature” — a notion developed precisely to study the political representations of literature rather than to define or promote a category of “political literature” (Denis 2006 ; Hamel 2014 ; recently discussed in academic research : Amir, Huppe & Jeusette 2025). Nor does the article provide a justification for the shift from the broader category of "political literature" to that of the novel — in other words, for the choice to concentrate its theoretical inquiry on the novel as such. I am also struck by the lack of clarity regarding the geographical and cultural grounding of the reflection in this introduction.

Is the argument persuasive and supported by appropriate evidence?

Partly

Is the work clearly and cogently presented?

Yes

Is the topic of the essay discussed accurately in the context of the current literature?

Yes

Does the essay contribute to the cultural, historical, social understanding of the field?

Partly

Reviewer Expertise:

Contemporary literature, literature and politics, crime fiction,

I confirm that I have read this submission and believe that I have an appropriate level of expertise to confirm that it is of an acceptable scientific standard.

# Reviewer report: Reviewer response for version 1

**DOI:** 10.21956/openreseurope.21543.r56404

**Published:** 2025-08-23

**Authors:** Alina Bako

**Affiliations:** 1 Lucian Blaga University of Sibiu, Sibiu, Romania

The article offers a comprehensive exploration of the concept of the "political novel” and critically examines the term by addressing its methodological issues, its evolving nature, and its significance in literary studies. The authors engage with a range of theoretical perspectives, drawing on thinkers like Franco Moretti, Jacques Rancière,  Irving Howe, Stuart Hall, William Sharpe, Claude Lefort, Pierre Rosanvallon, Immanuel Nover, Stefan Neuhausand, Michael Navratil, Zvonimir Glavaš, Jonathan Cullerto interrogate the interplay between politics, literature, and the novel as a genre.

 The article is structured in two parts: a theoretical discussion of the political novel and case studies of specific works, offering a nuanced approach to understanding this elusive category. The results of the Caponeu project are discussed, including the presentation of an interactive map resulting from the articles and research conducted within it.

 The article emphasizes the importance of historicizing and contextualizing the political novel. It recognizes that the political is not a universal or static category but varies across time, geography, and ideology.

 The second part of the article, with its analysis of novels like Vladan Desnica’s novel *Zimsko ljetovanje* ( *The Winter Summer Vacation*, 1950), Samuel Beckett’s *Molloy* (1951), Michał Witkowski’s *Lubiewo* (2005) and Pierre Michon’s *Les Onze* ( *The Eleven*, 2009) to demonstrates the practical application of the theoretical framework. These case studies illustrate how novels not traditionally seen as political can be read through a political lens, revealing new interpretive possibilities.

 The selection of case studies, while insightful, is limited to a specific set of European novels. Including a wider range of texts from diverse cultural or geographical contexts could have strengthened the article’s claim to a global understanding of the political novel.

 By questioning the separation of literature from authorial intention and traditional politics, the article aligns with contemporary literary theory while pushing for a more inclusive understanding of what constitutes the political.

 We recommend the article for indexing, as it provides a viable overview of the political novel, proposing novel and clarifying perspectives. The theoretical literature is complex and coherently constructs the concept, as do the references to the proposed case studies.

Is the argument persuasive and supported by appropriate evidence?

Yes

Is the work clearly and cogently presented?

Yes

Is the topic of the essay discussed accurately in the context of the current literature?

Yes

Does the essay contribute to the cultural, historical, social understanding of the field?

Yes

Reviewer Expertise:

Literary Studies, World Literature, Critical Studies

I confirm that I have read this submission and believe that I have an appropriate level of expertise to confirm that it is of an acceptable scientific standard.
